# Effectiveness of herbal plants on rumen fermentation, methane gas emissions, *in vitro* nutrient digestibility, and population of protozoa

**DOI:** 10.14202/vetworld.2023.1477-1488

**Published:** 2023-07-19

**Authors:** Antonius Antonius, Roni Pazla, Ezi Masdia Putri, Windu Negara, Nursanti Laia, Muhammad Ridla, Sri Suharti, Anuraga Jayanegara, Sharli Asmairicen, Leni Marlina, Yoselanda Marta

**Affiliations:** 1 Research Center for Animal Husbandry, National Research and Innovation Agency (BRIN), Jl. Raya Jakarta Bogor Cibinong, 16915, Indonesia; 2 Department of Animal Nutrition, Faculty of Animal Science, Andalas University, Jl. Limau Manis, Padang, 25163, Indonesia; 3 State community Academy Nias Utara, Jl Lolofoso Lotu Kab. Nias Utara, 22853, Indonesia; 4 Department of Nutrition and Feed Technology, Faculty of Animal Science, IPB University, Jl. Agatis Kampus IPB Dramaga Bogor, 16680, Indonesia; 5 Research Center for Agroindustry, National Research and Innovation Agency (BRIN). Jl. Puspitek Tangerang Selatan, 15314, Indonesia; 6 Center for Breeding Superior Livestock and Forage for Animal Feed Padang Mengatas, Jl. Raya Payakumbuh-Lintau, KM.9 Pekan Sabtu, Payakumbuh, 26201, Indonesia

**Keywords:** herbal plants, *in vitro*, methane, protozoa

## Abstract

**Background and Aim:**

Herbal plants have the potential to reduce the population of metagonic bacteria and protozoa due to the bioactive compound contained in herbal plants. This study aimed to evaluate the effect of herbal plant supplementation on rumen fermentation characteristics, methane (CH_4_) gas emissions, *in vitro* nutrient digestibility, and protozoan populations.

**Materials and Methods:**

This study consisted of two stages. Stage I involved determining the potential of herbal plants to increase total gas production (Orskov and McDonald methods) and reduce the protozoan population (Hristov method). Three potential herbs were selected at this stage and used in Stage II as supplements in the palm kernel cake (PKC)-based diet (30% herbal plants + 70% PKC). Proximate and Van Soest analyses were used to determine the chemical composition. *In vitro* dry matter digestibility (IVDMD), organic matter (IVOMD), and rumen fermentation characteristics were determined using Theodorous method. Conway microdiffusion was used to determine ammonia concentration (NH_3_). Gas chromatography was used to determine the total and partial volatile fatty acid production.

**Results:**

The results of the first stage showed that seven herbal plants (*Moringa oleifera*, *Rhodomyrtus tomentosa*, *Clerodendron serratum*, *Curcuma longa* Linn., *Urena lobata*, *Uncaria*, and *Parkia timoriana*) significantly differed in terms of total gas production (p < 0.05). Herbal plants can increase gas production and reduce protozoan populations. The highest total gas production was observed using *P. timoriana*, *M. oleifera*, and *C. longa* Linn. *Moringa oleifera* plants were the most effective in lowering protozoa population. In Stage 2, the supplementation of herbal plants in PKC-based-diet significantly increased IVDMD, that was ranged from 56.72% to 65.77%, IVOMD that was ranged from 52.10% to 59.54%, and NH_3,_ that was ranged from 13.20 mM to 17.91 mM. Volatile fatty acid partial and total gas production potential and CH_4_ gas emissions were also significantly different from those of the control (p < 0.05).

**Conclusion:**

Supplementation of *M. oleifera*, *C. longa* Linn., and *P. timoriana* in ruminant diet effectively increased total gas production, IVDMD percentage, and IVOMD, and reduced CH_4_ gas emissions and protozoa populations during rumen fermentation.

## Introduction

Global warming is a major environmental problem. Carbon dioxide (CO_2_), methane (CH_4_), nitrogen oxides, and chlorofluorocarbons are the primary contributors to global warming. Methane is the result of the anaerobic fermentation of structural and non-structural carbohydrates by methanogens (methanobacteria) in the rumen of ruminants and is released into the atmosphere by belching (burping). Methane emitted from the rumen corresponds to ruminant energy losses that vary between 7% and 12% of the energy expended [1, 2]. In addition, the CH_4_ produced by ruminants contributes significantly to global warming. About 43% of greenhouse gas emissions come from livestock in the form of CH_4_ [3].

Protozoan populations in the rumen are directly proportional to CH_4_ production. This means that fewer protozoa in the rumen produce less CH_4_ gas. Thus, CH_4_ gas emissions can be reduced by providing protozoan defaunator substances, such as saponins, to the population of fiber-digesting bacteria [4]. In addition, tannins have been shown to reduce the number of ruminal methanogens by inhibiting the digestion of fiber components, thereby reducing the production of H_2_ which can reduce CH_4_ emissions [5–7].

One approach to minimizing CH_4_ emissions in ruminants is through a feeding strategy. Many feeding management approaches have been used to reduce CH_4_ production such including quality forage, increasing the amount of concentrate, supplementation of fats and oils, and addition of plant secondary metabolites (active substances) [8]. Using herbal plants in the diet can reduce CH_4_ gas production [8–10]. *Azadirachta indica*, *Moringa oleifera*, *Ocimum gratissimum*, garlic (*Allium sativum*), ginger (*Zingiber officinale*), and onion leaflets (*Allium cepa*) can affect ruminal fermentation and reduce CH_4_ production in sheep [11]. Garlic and coconut oils can reduce the number of CH_4_-producing bacteria [12]. *Punica granatum* and *Tecomella undulata* herb supplementation can help improve enteric CH_4_ production, thereby improving the growth performance of ruminants [13].

The use of local herbal plants in diets to reduce CH_4_ gas production and suppress protozoan populations has not yet been widely reported. The active substances in these plants include tannins, saponins, and flavonoids. Tannins can reduce CH_4_ emissions by reducing methanogen populations in the rumen [6] and inhibiting the digestion of dietary fiber components, thereby reducing H_2_ production [7]. Thus, this study aimed to determine the effect of herbal plant supplementation on rumen fermentation characteristics, CH_4_ gas emission, *in vitro* nutrient digestibility, and protozoan population. We hypothesized that these herbs have the potential to maintain *in vitro* fermentation characteristics and nutrient digestibility and depress the protozoan population, leading to a reduction in CH_4_ gas production.

## Materials and Methods

### Ethical approval

Ethical approval was not required because we did not use live animals in this study.

### Study period and location

This study was conducted from May 2016 to September 2016 at the Institute of Feed Science and Technology, Faculty of Animal Husbandry, Bogor Agricultural University, Bogor, West Java, Indonesia and Institute of Food and Nutrition Research Center, University of Gadjah Mada, Yogyakarta, Indonesia

### Stage I

#### Sample preparation

This stage aimed to select the potential herbal plants to increase gas the total gas production and reduce the protozoan population. The herbal plants included *M. oleifera*, *Rhodomyrtus tomentosa*, *Clerodendron serratum*, *Curcuma longa* Linn., *Urena lobata*, *Uncaria*, and *Parkia timoriana*. These herbal plants were collected from the medicinal plant garden of the Materia Medica Batu Herbal Laboratory, East Java, Indonesia. The position of this area is 7° 52′ S 112° 31′ E with an altitude of 800 m–2000 m above sea level and the average temperature is 11°C–19°C. The average rainfall is 298 mm/month, which occurs approximately 6 times a month. These plants were identified by Siti Mudaliana, S.Si, M.Sc, a botanist from the Materia Medica Batu Herbal Laboratory in East Java, Indonesia. Specimens (*M. oleifera:* MMB-225Mol, *C. longa* Linn.: MMB-268Clo, *P. timoriana*: MMB-214Pro, *Cleodendron serratum*: MMB-421Cse) were deposited in the herbarium of Materia Medica Batu Herbal Laboratory, East Java, Indonesia. The leaves were selected as the experimental sample, oven-dried at 60°C for 24 h, and milled through a 1 mm sieve.

#### In vitro incubation

The herbal plants were incubated using the Theodorou method [14]. The rumen fluid was filtered using a filter cloth, placed in a thermos bottle, and transported to the laboratory. The rumen fluid (100 mL) and buffer (75 mL) were added to the bottles. The substrate and the mixture of buffer and rumen fluid in the bottle were incubated anaerobically by pumping CO_2_ gas into the bottle covered with rubber and aluminum foil. The samples were incubated in a 39°C water bath.

#### Gas velocity and total gas production measurement

After incubation, the gas production rates and total gas production were measured at 2, 4, 8, 12, 24, 36, 48, 60, and 72 h. A 60 mL capacity plastic syringe was used for this measurement. The total amount of gas produced was pushed inside the syringe. After the gas had pushed the syringe plunger, the reading on the syringe scale was recorded. The gas production rate and total gas production were estimated using the Orskov equation [15]. The equation is as follows:

p = a + b (1 – e^-ct^)

Note:

p: Cumulative gas production at time t hours

a: Gas production of soluble fraction

b: Gas production of unsoluble fraction that can be fermented

c: Speed of gas production (mL/h)

t: Incubation time (h).

#### Protozoa population measurement

One milliliter of incubated sample was added to 1 mL of trypan blue formalin saline, prepared from a mixture of 100 mL of 4% formalin and 0.9% physiological NaCl solution. Then, two drops of the mixture were added to a 0.1 mm thick counting chamber. The minimum area of each of 16 boxes was 0.0625 mm^2^. Calculations of the protozoan population were performed under a microscope at 10× magnification. The protozoan population was calculated using the following formula:


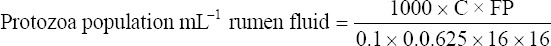


Where C is the number of protozoa counted and FP is the dilution factor.

### Stage II

#### Sample preparation

This study examined the effect of herbal plant supplementation in feed consisting of 70% palm kernel cake (PKC) and 30% herbal plants. Based on the results of Stage 1, the three selected herbal plants were *M. oleifera*, *C. longa* Linn., and *P. timoriana*. The selected plants showed the highest gas production and protozoan population reduction. This experiment used a randomized block (RBD) design with four replicates performed in triplicate. The experimental diet in Stage II was as follows:

- R1: 100% PKC
- R2: 70% PKC + 30% *M. oleifera* (MO)
- R3: 70% PKC + 30% *C. longa* Linn. (CL)
- R4: 70% PKC + 30% *P. timoriana* (PT)
- R5: 70% PKC + 15% MO + 15% CL
- R6: 70% PKC + 15% MO + 15% PT
- R7: 70% PKC + 15% CL + 15% PT
- R8: 70% PKC + 10% MO + 10% CL + 10% PT.

The chemical composition of each ingredient and treatment diet is given in Tables-1 and 2, respectively. The variables observed in Stage II were the degradability of dry matter and organic matter, ammonia (NH_3_) concentration, rumen fluid pH, partial volatile fatty acid (VFA) concentration (acetate, propionate, and butyrate), gas production rate, total gas production, total protozoa population, and production of CH_4_ *in vitro*. The rumen fluid for *in vitro* incubation was collected in the morning before fistula-fried Frisian Holstein dairy cows were fed at the stables of the Animal Research Institute (Balitnak) Ciawi, Bogor, Indonesia.

**Table-1:** Proximate analysis of feed ingredients.

Nutrition	Feed ingredient			

	PKC	*M. oleifera*	*C. longa* Linn.	*P. timoriana*
DM (%)	90.8	86.5	84.3	91.6
Ash (% DM)	6.61	14.8	6.83	5.50
CP (% DM)	14.5	29.1	8.43	20.1
EE (% DM)	6.7	3.9	7.63	9.10
CF (% DM)	26.2	6.9	10.5	10.4
NDF (% DM)	61.7	14.9	60.1	23.8
ADF (%)	39.2	10.7	9.04	13.3
Lignine (%)	10.9	td*	td*	td*
Cellulose (%)	26.3	td*	td*	td*

td*=Not tested, DM=Dry matter, CP=Crude protein, EE=Extract ether, CF=Crude fiber, NDF=Neutral detergent fiber, ADF=Acid detergent fiber, PKC=Palm kernel cake, *M. oleifera*=*Moringa oleifera*, *C. longa* Linn.=*Curcuma longa* Linn., *P. timoriana*=*Parkia timoriana*

**Table-2:** Nutrient ingredients of experimental diets.

Treatment	Nutrition (% DM)			

	CP	CF	EE	Ash
R1	14.5	26.2	6.77	6.61
R2	18.9	20.4	5.91	9.08
R3	12.7	21.5	7.03	6.68
R4	16.2	21.4	7.47	6.28
R5	15.8	20.9	6.46	7.87
R6	17.6	20.9	6.69	7.68
R7	14.5	21.5	7.25	6.48
R8	15.9	21.1	6.80	7.34

R1=100% palm kernel cake, R2=70% palm kernel cake + 30% *Moringa oleifera*, R3=70% palm kernel cake + 30% *Curcuma longa* Linn., R4=70% palm kernel cake + 30% *Parkia timoriana*, R5=70% palm kernel cake + 15% *Moringa oleifera* + 15% *Curcuma longa* Linn., R6=70% palm kernel cake + 15% *Moringa oleifera* + 15% *Parkia timoriana*, R7=70% palm kernel cake + 15% *Curcuma longa* Linn. + 15% *Parkia timoriana*, R8=70% palm kernel cake + 10% *Moringa oleifera* + 10% *Curcuma longa* Linn. + 10% *Parkia timoriana*. DM=Dry matter, CP=Crude protein, CF=Crude fiber, EE=Extract ether

#### Chemistry analysis of samples

Selected plant samples were then analyzed for qualitative phytochemical (Table-3) and nutrient contents using proximate analyses [16]. Neutral detergent fibers and acid detergent fiber were analyzed using Van Soest analysis [17].

**Table-3:** Qualitative photochemical content of *M. oleifera*, *C. longa* Linn., and *P. timoriana*.

Variable	*M. oleifera*	*C. longa* Linn.	*P. timoriana*
Flavanoids	+	+	-
Alkaloids	-	-	-
Tannins	+	-	-
Saponins	+	+	+
Steroids	+	-	+
Triterpenoids	-	+	-
Quinone	-	+	-

*M. oleifera=Moringa oleifera, C. longa* Linn.=*Curcuma longa* Linn*., P. timoriana=Parkia timoriana*

#### In vitro nutrient digestibility measurements

*In vitro* dry matter digestibility (IVDMD) and *in vitro* organic matter digestibility (IVOMD) were determined using the methods described by Tilley and Terry [18]. Fermentation tubes were loaded with 0.5 g of sample, a mixture of 10 mL of ruminal fluid, and 40 mL of McDougal’s solution. The samples were anaerobically incubated by pumping CO_2_ gas for 30 s and then covered with rubber and aluminum foil. Samples were incubated in a shaker at 39°C for 48 h. After 48 h, 2–3 drops of HgCl_2_ were added to the fermentation tube to stop the microorganism activity. The residue and supernatant were separated by centrifugation at 1792× *g* for 10 min. Separation was continued by adding 50 mL of 0.2% pepsin HCl solution and then centrifuging at 1792 × *g* for 15 min. The mixture was incubated for an additional 48 h with the rubber stopper removed. The residue was filtered through a Whatman #1 filter paper 41 (GE Healthcare, Buckinghamshire, Britain) (known weight) using a vacuum pump. The residue on the filter paper was placed in a porcelain cup and oven-dried at 105°C for 24 h. After 24 h, the samples were weighed to determine their dry matter content. In addition, the material in the porcelain cup was fired at a temperature of 600°C or fired in an electric furnace for 6 h to determine the content of organic matter. The fermentation residue containing no feed components was used a blank.


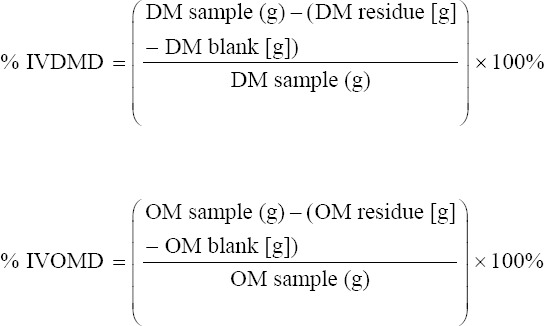


Where: IVDMD = *In vitro* dry matter digestibility

IVOMD = *In vitro* organic matter digestibility

DM = Dry matter

OM = Organic matter

#### Ammonia and pH measurement

Measurement of NH_3_ was performed using the Conway, microdiffusion method [19]. The supernatant resulting from 4 h of incubation was centrifuged at – 1372× *g* for 15 min. Then, 1 mL was taken and placed in the Conway glass, 1 mL of saturated Na_2_C_3_ 0.005 M solution also placed in the Conway glass, and in the middle Conway glass was filled with 1 mL of indicated boric acid. Conway’s glass, which has a lid smeared with Vaseline, is tightly closed to make it airtight, following Finlay’s study described by Finlay’s *et al*. [20]. The supernatant and Na_2_Co_3_ were mixed evenly until the color changed from blue to red. After incubation for 24 h, indicated boric acid was titrated with H_2_SO_4_ 0.005 M. The concentration of NH_3_ was calculated using the following formula:


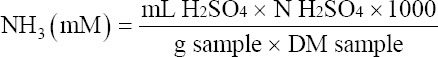


The pH of *in vitro* rumen fluids was measured using a Jenway Model 3505 pH meter, Keison Products, England. Before use, the pH meter was calibrated using a solution of pH 7.

#### Volatile fatty acids concentration measurement

Fermented samples incubated for 4 h were placed in an Eppendorf tube (Wujiang, Jiangsu Province, China), and the pH was lowered to 3 by adding one drop of concentrated H_2_SO_4_ solution to each sample. The concentrated acid was added aimed to stabilize the sample for gas measurements. Measurement of total and partial VFA production (acetic acid, propionate, and butyrate) was performed using Gas Chromatography (GC 8A, Shimadzu Crop, Kyoto, Japan) with a column containing 10% SP-1200, 1% H_2_PO_4_ at 80/100 Cromosorb WAW (Imerys Minerals, California Inc, USA). First, 1 μL of the standard solution was injected into the GC, and then, 1 μL of the incubated sample was injected. The results of the analysis were read on the chromatogram. The VFA concentrations in the samples were calculated using the following formula:


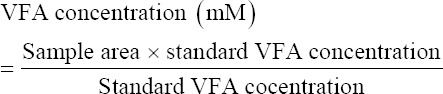


### Determination of CH_4_ emissions

CH4 gas emissions were measured at the same incubation time as the measurements of total gas production. CH_4_ gas emissions were measured using the CO_2_ trap method with an alkaline trapping agent in the form of NaOH, following the Fievez method described by Fievez *et al*. [21]. CH_4_ was measured after measuring the total gas production. The needle was removed after determining the total gas volume in the syringe. The channel at the end of the syringe was connected to the inlet of a 5 M NaOH solution in an Erlenmeyer flask, while the Erlenmeyer outlet containing NaOH was connected to the inlet of a 10 mL scale syringe attached to the burette with the tip down.

After the syringe was connected to the inlet of the NaOH solution, the syringe rod was slowly pushed until the total gas produced passed through the NaOH solution. At the time of total gas production, it was trapped by the NaOH solution, while the gas component in the form of CH_4_ passed through the Erlenmeyer outlet and entered the 10 mL syringe inlet. The CH_4_ gas volume (mL) was determined using manual balance readings.

### Statistical analysis

All data are presented descriptively with one standard deviation. This study adopted a RBD design. In Stage I, experiment with the seven herbal plants was repeated 3 times to determine the chemical composition of the feed ingredients, total gas production, gas production rate, and number of protozoa. Stage II included eight treatments with three replicates based on the timing of rumen fluid collection for *in* *vitro* testing. The data were analyzed using analysis of variance, and when there were significant differences; further, Duncan’s tests were performed using the Statistical Package for the Social Science version 16.0 (IBM Corp., NY, USA).

## Results

### Stage I (seven herbal plant screening)

#### Gas production rate, total gas production, and protozoa population

**Figure-1:**
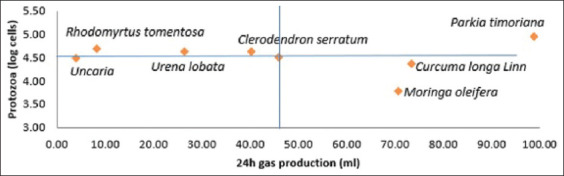
Protozoa population and gas amount.

Figure-1 provides information on the effect of the herbal plants screened on the protozoan population and total gas production. The total gas production between each plant was significantly different (p < 0.05). The highest gas production was observed in the *P. timoriana* and the lowest was observed in *Uncaria*. Gas production of 98.83, 73.50, 70.67, 40.33, 26.50, 8.33, and 4.00 mL was observed in *P. timoriana*, *C. longa* Linn., *M. oleifera*, *U. lobata*, *C. serratum*, *U. lobata*, *R. tomentosa*, and *Uncaria*, respectively.

The lowest number of protozoa was observed using *M. oleifera*, which was significantly different from that observed using the other plants (p < 0.05). The number of protozoa in *M. oleifera* was the lowest compared to other plants. Stage I aimed to select potential herbal plants with high total gas production and low protozoan population. Thus, the results of the first phase of the study showed that the three best plants for high total gas production with low protozoan counts were *P. timoriana*, *C. longa* Linn., and *M. oleifera*. These herbal plants were used in Stage II as feed supplementation in the PKC-based diet.

### Stage II (*in vitro* evaluation of herbal supplementation on a diet)

#### In vitro dry matter digestibility and IVOMD

Treatment with dietary herbal plants had a significant effect on IVDMD. The addition of herbal plants significantly increased the IVDMD compared to that in control (p < 0.05). The addition of 30% *M. oleifera* (R2) or a mixture of herbal plants containing 15% *M. oleifera* and 15% *P. timoriana* (R5) had the same effect on IVDMD (Table-1). A decrease in IVDMD was observed in the addition of a mixture of herbal plants containing 10% *M. oleifera*, 10% *C. longa* Linn., or 10% *P. timoriana* (R8) (Table-4). *In vitro* dry matter digestibility value obtained using 10% *M. oleifera* was comparable to those obtained using 15% *C. longa* Linn. (R4) or a mixture of both (R7) (Table-4). The IVDMD value obtained using herbal plants up to 30% in the diet in this study ranged from 56.72% to 65.77% (Table-4). The average of IVDMD obtained using 30% *M. oleifera* (R2) was 63.82%, and that obtained using a mixture of herbal plants containing 15% *M. oleifera* and 15% *P. timoriana* was 65.77%. As a mixture herbal plants-PKC, the lowest IVDMD was obtained using 10% *M. oleifera*, 10% *C. longa* Linn., and 10% *P. timoriana* (R8). The increase in IVDMD was directly proportional to IVOMD. The addition of herbal plants significantly increased IVOMD compared with the control (p < 0.05). However, the IVOMD values obtained using different herbal plants were not significantly different from each other, but were significantly different from that of the control (Table-4). Based on the data from IVOMD, we hypothesized that the herbal plants had the same quality. In addition, the herbal plants did not interfere with microbial activity during fermentation. Therefore, they not negatively affect the digestion of organic matter. *In vitro* organic matter digestibility values in this study ranged from 52.10% to 59.54% (Table-4).

**Table-4:** *In vitro* nutrient digestibility and rumen fermentation profile of experimental diets.

Treatment	Variable			

	IVDMD (%)	IVOMD (%)	NH_3_ (mM)	pH
R1	42.84^a^ ± 3.53	34.77^a^ ± 4.04	11.71^a^ ± 0.76	6.80 ± 0.06
R2	63.82^c^ ± 3.25	57.74^b^ ± 8.34	13.20^b^ ± 1.46	6.82 ± 0.08
R3	59.34^bc^ ± 6.61	58.48^b^ ± 8.61	13.30^b^ ± 1.49	6.77 ± 0.05
R4	62.17^bc^ ± 9.79	53.84^b^ ± 13.04	13.23^b^ ± 1.63	6.75 ± 0.12
R5	65.77^c^ ± 5.94	52.10^b^ ± 5.30	17.07^c^ ± 2.19	6.63 ± 0.12
R6	65.16^c^ ± 4.46	59.54^b^ ± 2.70	17.02^c^ ± 2.47	6.70 ± 0.13
R7	60.56^b^ ± 3.48	56.68^b^ ± 5.80	17.14^c^ ± 2.11	6.73 ± 0.14
R8	56.72^b^ ± 3.66	53.15^b^ ± 7.77	17.91^c^ ± 1.57	6.68 ± 0.04

Different superscripts in the same column show significant differences (p<0.05). R1=100% palm kernel cake, R2=70% palm kernel cake + 30% *Moringa oleifera*, R3=70% palm kernel cake + 30% *Curcuma longa* Linn., R4=70% palm kernel cake + 30% Parkia timoriana, R5=70% palm kernel cake + 15% *Moringa oleifera* + 15% *Curcuma longa* Linn., R6=70% palm kernel cake + 15% *Moringa oleifera* + 15% Parkia timoriana, R7=70% palm kernel cake + 15% *Curcuma longa* Linn. + 15% Parkia timoriana, R8=70% palm kernel cake + 10% *Moringa oleifera* + 10% *Curcuma longa* Linn. + 10% Parkia timoriana. IVDMD=*In vitro* dry matter digestibility, IVOMD=*In vitro* organic matter digestibility, NH_3_=Ammonia

### The concentration of NH_3_ and pH value of rumen fluid

Table-4 provides information on the effect of herbal plant supplementation on NH_3_ concentration. The addition of herbal plants increased the production of NH_3_ compared with the control (p < 0.05). The average of NH_3_ in this study ranged from 11.71 mM to 17.91 mM, which was within the normal range. This indicates that herbal plants did not interfere with rumen microbial activity. The highest NH_3_ concentration was found on addition of 10% *M. oleifera*, 10% *C. longa* Linn., and 10% *P. timoriana* (R8) which were 17.91 mM; R5, R6, and R7 gave results identical to those of R8. The lowest value was observed in the absence of herbal plant supplementation (R1), which was 11.71 mM.

The pH values are listed in Table-4. The pH values during incubation with the eight experimental diets ranged from 6.63 to 6.82. However, the difference was not statistically significant. The highest pH value was found in R2 treatment with 30% *M. oleifera*, namely, 6.82, and the lowest value in R5 treatment with 15% *M. oleifera* and 15% *C. longa* Linn., namely, 6.63. However, the pH values among all ration treatments were not significantly different.

### Total VFA and partial VFA concentration

The VFA (acetate, propionate, and butyrate acid) concentrations are presented in Table-5. The total VFA concentration of in the control fluid was 35.80 mM, which was not significantly different from the total VFA concentrations in the treated rumen fluid (p < 0.05). The mean total VFA in this study ranged from 34.94 to 44.71 mM. The highest total VFA concentration was found in R7, which contains 15% *C. longa* Linn. and 15% *P. timoriana*, namely, 44.71 mM and the lowest was in R3, which contains 30% *C. longa* Linn. The individual concentrations (acetate, propionate, and butyrate acid) were significantly different (p < 0.05).

**Table-5:** Partial VFA and total VFA of experimental diets.

Treatment	Variable			Total VFA (mM)

	Acetate acid (%)	Propionate acid (%)	Butyrate acid (%)	
R1	64.27^ab^ ± 6.71	21.45 ± 2.24	14.28 ± 1.20	35.80 ± 10.06
R2	64.87^a^ ± 8.49	20.98 ± 2.63	14.15 ± 2.00	38.68 ± 12.94
R3	61.51^a^ ± 8.61	22.98 ± 2.94	15.51 ± 2.06	34.94 ± 13.41
R4	61.88^ab^ ± 10.72	22.65 ± 3.07	15.47 ± 2.69	38.40 ± 16.23
R5	62.29^ab^ ± 3.67	21.93 ± 1.47	15.77 ± 1.28	41.99 ± 6.15
R6	62.85^ab^ ± 5.39	22.02 ± 1.32	15.13 ± 1.63	41.60 ± 8.21
R7	63.99^ab^ ± 1.81	21.98 ± 0.71	14.02 ± 1.10	44.71 ± 1.97
R8	64.25^ab^ ± 2.02	21.04 ± 0.77	14.71 ± 0.91	40.00 ± 3.33

Different superscripts in the same column show significant differences (p<0.05). R1=100% palm kernel cake, R2=70% palm kernel cake + 30% *Moringa oleifera*, R3=70% palm kernel cake + 30% *Curcuma longa* Linn., R4=70% palm kernel cake + 30% *Parkia timoriana*, R5=70% palm kernel cake + 15% *Moringa oleifera* + 15% *Curcuma longa* Linn., R6=70% palm kernel cake + 15% *Moringa oleifera* + 15% *Parkia timoriana*, R7=70% palm kernel cake + 15% *Curcuma longa* Linn. + 15% *Parkia timoriana*, R8=70% palm kernel cake + 10% *Moringa oleifera* + 10% *Curcuma longa* Linn. + 10% *Parkia timoriana*. VFA=Volatile fatty acid

### Average gas production and gas production rate per hour

The average gas production and gas production rates per hour are listed in Table-6. The addition of herbal plants significantly affected the average gas production (p < 0.05), whereas the gas production rate was not significantly different among the treatments (p > 0.05). R7 (15% *C. longa* Linn. and 15% *P. timoriana*) had the highest total gas production, while R2 (30% *M. oleifera*) had the lowest average gas production. Table-6 shows that the average gas production using R8 was lower than that R7, but higher than that using other herbal plants (p < 0.05). This indicates increased gas production when a herbal plant combination was added. There were no significant differences (p > 0.05) in gas production rates per hour among the herbal plant treatments. Herbal supplementation was comparable to the control (Table-6).

**Table-6:** Average value of gas production potential and gas production rate per hour of experimental diets.

Treatment	Average gas production potential (mL)	Gas production rate per hour (mL/h)
R1	98.01^a^ ± 13.21	0.051 ± 0.006
R2	134.84^b^ ± 5.25	0.054 ± 0.002
R3	141.68^c^ ± 10.34	0.051 ± 0.003
R4	146.20^d^ ± 3.61	0.054 ± 0.006
R5	139.76^bcd^ ± 4.37	0.053 ± 0.005
R6	138.84^bc^ ± 5.02	0.054 ± 0.002
R7	154.00^f^ ± 1.30	0.053 ± 0.006
R8	147.57^e^ ± 5.77	0.053 ± 0.008

Different superscripts in the same column show significant differences (p<0.05). R1=100% palm kernel cake, R2=70% palm kernel cake + 30% *Moringa oleifera*, R3=70% palm kernel cake + 30% *Curcuma longa* Linn., R4=70% palm kernel cake + 30% *Parkia timoriana*, R5=70% palm kernel cake + 15% *Moringa oleifera* + 15% *Curcuma longa* Linn., R6=70% palm kernel cake + 15% *Moringa oleifera* + 15% *Parkia timoriana*, R7=70% palm kernel cake + 15% *Curcuma longa* Linn. + 15% *Parkia timoriana*, R8=70% palm kernel cake + 10% *Moringa oleifera* + 10% *Curcuma longa* Linn. + 10% *Parkia timoriana*

### Methane emissions and protozoa populations

The gas emissions and protozoan populations are listed in Table-7. The results showed that the control (R1) combined with one of R2, R3, and R4 or the combination of herbal plants (R5, R6, R7, and R8) significantly reduced CH_4_ gas production (p < 0.05). The average CH_4_ production for each treatment was as follows: R1 = 140 ± 40.88 mL CH_4_/g DOM, R2 = 116 ± 25.68 mL CH_4_/g DOM, R3 = 113 ± 30.51 mL CH_4_/g DOM, R4 = 113 ± 27.54 mL CH_4_/g DOM, R5 = 114 ± 20.11 mL CH_4_/g DOM, R6 96 ± 9.35 mL CH_4_/g DOM, R7 = 108 ± 15.70 mL CH_4_/g DOM, and R8 = 111 ± 26.30 mL CH_4_/g DOM.

**Table-7:** Average CH_4_ gas production and protozoa populations of experimental diets.

Treatment	CH_4_ (mL/g DOM)	Protozoa (log CFU/mL)
R1	140^b^ ± 40.88	4.42 ± 0.123
R2	116^a^ ± 25.68	4.319 ± 0.069
R3	113^a^ ± 30.51	4.358 ± 0.069
R4	113^a^ ± 27.54	4.358 ± 0.069
R5	114^a^ ± 20.11	4.389 ± 0.107
R6	96^a^ ± 9.35	4.264 ± 0.143
R7	108^a^ ± 15.70	4.303 ± 0.164
R8	111^a^ ± 26.30	4.319 ± 0.068

Different superscripts in the same column show significant differences (p < 0.05). R1=100% palm kernel cake, R2=70% palm kernel cake + 30% *Moringa oleifera*, R3=70% palm kernel cake + 30% *Curcuma longa* Linn., R4=70% palm kernel cake + 30% *Parkia timoriana*, R5=70% palm kernel cake + 15% *Moringa oleifera* + 15% *Curcuma longa* Linn., R6=70% palm kernel cake + 15% *Moringa oleifera* + 15% *Parkia timoriana*, R7=70% palm kernel cake + 15% *Curcuma longa* Linn. + 15% *Parkia timoriana*, R8=70% palm kernel cake + 10% *Moringa oleifera* + 10% *Curcuma longa* Linn. + 10% *Parkia timoriana*. CFU=Colony-forming unit, CH_4_=Methane, DOM=Digestibility of organic matter

The protozoan population results presented in Table-7 show that the control treatment (R1) combined with one of the herbal plants R2, R3, R4, R6, R7, and R8 or combinations had no significant effect on the decrease in the protozoan population (p > 0.05).

## Discussion

### Gas production rate, total gas production, and protozoa population

Total gas production and protozoan populations significantly differed among the experimental treatments (p < 0.05). As shown in Figure-1, the highest total gas production was observed using *P. timoriana*, followed by *M. oleifera* and *C. longa* Linn. and the lowest was observed using *Uncaria* and *R. tomentosa*. This is consistent with the findings of Faniyi *et al*. [11], who found that several herbal plants significantly increased anaerobic gas production. The gas production rate of anaerobic fermentation increased linearly with the increase of IVOMD. *In vitro* organic matter digestibility reflects the energy and fermentable substrates available to ruminants and the microbial rumen for degradation. The present study showed that the combination of *P. timoriana* and *M. oleifera* produced the highest IVOMD, which is consistent with the finding of high total gas production these herbal plants.

The number of protozoa in samples treated with *M. oleifera* was the lowest and significantly different (p < 0.05) compared to other plants. *Moringa oleifera* contains saponins and tannins as defecation agents that can suppress protozoa populations so that bacteria can develop in the rumen. Saponins from the leaves of herbal plants can increase the efficiency of the fermentation process by reducing protozoan populations in the rumen and their predatory properties against bacteria [22–24]. This is in agreement with the study by Okoruwa and Aidelomon [25], who found that supplementation of sheep and goat diets with ginger powder significantly reduced the total protozoa. According to Jouany [26], as much as 70% of methanogenic bacteria are in symbiosis with protozoa. Except *M. oleifera*, the other herbal plants had no effect on the number of protozoans. Thus, the results of the first phase of the study showed that the three best plants for high total gas production with low protozoan counts were *P. timoriana*, *C. longa* Linn., and *M. oleifera*.

### *In vitro* dry matter digestibility and IVOMD

This study reported that adding herbal plants significantly affected IVDMD compared with the control (p < 0.05). This indicates that herbal plants used are a source of high levels of degradable protein (protein required by rumen microbes), which stimulates rumen microbial growth and activity for improved feed digestion [26–29]. A previous study reported by Abd El Tawab *et al*. [30] found that supplementation with herbal plants increased IVDMD and IVDOM values compared to control feeds.

The highest IVDMD was observed for R5, which contains of 15% *M. oleifera* and 15% *C. longa* Linn. (Table-4). The IVDMD value of the experimental herbal plants was significantly higher than that of the control. *Moringa* *oleifera* is a plant that is effective in inhibiting the growth or activity of pathogenic microorganisms because it contains saponins, flavonoids, tannins, alkaloids, and phenols [31], thereby resulting in the growth of non-pathogenic microbes and improvement in nutrient digestion. In addition, the previous study by Ebeid *et al*. [32] showed that *M. oleifera* contains proteins that are easily decomposed which can increase the growth of rumen microbes. In this study, IVDMD was increased up to 30% (range: 56.72%–65.77%). Similarly, the previous study by Leitanthem *et al*. [33] also reported an increase in IVDMD in a diet containing *M. oleifera*. Antibacterial and protozoan reduce CH_4_ gas production, while increasing acetate production, which increases carbohydrate digestion in ruminants.

The increase in IVDMD was directly proportional to IVOMD. The addition of herbal plants significantly increased IVOMD compared to that of the control (p < 0.05). However, the IVOMD values did not differ significantly between the different herbal plants (Table-4). We hypothesized that the herbal plants have the same quality. In addition, the herbal plants did not interfere with microbial activity during fermentation. Therefore, they did not interfere with the digestion of organic matter. *In vitro* organic matter digestibility values obtained using different herbal plants in this study ranged from 34.77% to 59.54%. In accordance with Leitanthem *et al*. [33], an increase in IVOMD has also been reported in diets containing herbal plants. This indicates that herbal plants do not interfere with rumen microbes during the digestion of organic matter. In addition, herbal plants do not alter the nutritional composition of the diet, which can decrease in the digestibility of ration nutrients.

### Ammonia concentration and pH value

The addition of herbal plants increased the production of NH_3_ compared to the control (p < 0.05). This indicated that herbal plants increase microbial activity during protein degradation in the rumen. These results are supported by a previous study by Morsy *et al*. [34] that showed that *M. oleifera* contains easily degradable proteins that can increase the growth of rumen microbes. An increase in degraded protein will increase the production of NH_3_ in the rumen [35].

NH_3_ concentrations in this study ranged from 11.71 mM to 17.91 mM. According to previous research by McDonald *et al*. [36], the optimum range of NH_3_ in the rumen is 85 mg/l–300 mg/l or 6 Mm–21 Mm. This indicates that the herbal plants do not interfere with rumen microbial activity. The N-NH_3_ derives from the degradation of feed protein [37] and the degradation of microbial protoplasm, especially protozoa. Protozoa regulate the rate of nitrogen movement in the rumen and supply soluble proteins to maintain bacterial growth [38]. The NH_3_ concentration reflects the degradation of feed and endogenous proteins by rumen microbes [28]. If the feed lacks protein or is high in proteins that escape degradation, the rumen NH_3_ concentration will be low (lower than 50 mg/L or 3.57 mM), and the growth of rumen organisms will be slow. Conversely, if protein degradation is faster than microbial protein synthesis, NH_3_ accumulates and exceeds its optimum concentration [39]. The main factor affecting the use of NH_3_ in rumen fluid is the availability of crude fiber for rumen microorganisms [40].

An increase in protein content in the ration will increase the NH_3_ content of the rumen because 60% of feed protein will be converted into ammonia, whereas 40% will be passed on to the abomasum and small intestine to be digested, absorbed, and partially excreted in the feces [41]. High concentrations of N-NH_3_ in the rumen indicate a faster process of feed protein degradation than microbial protein formation, and ammonia accumulates in the rumen.

The pH values in this study were not significantly different (p > 0.05). The incubation time (pH measured 24 h after incubation) affected the availability of feed ingredients to be fermented by microbes. The high and low pH of rumen fluid is one of the determining factors for efficient fermentation. A previous study by Puniya *et al*. [42] stated that rumen microbial activity requires certain pH conditions related to the changing rumen conditions, and normal rumen pH to maintain normal rumen metabolism ranges from 6.0 to 7.0. The degree of acidity in the rumen can affect the population of active microorganisms during fermentation [43].

The activity of cellulolytic bacteria is inhibited if the pH of the rumen fluid is below 6.2, and their activity is optimal in the rumen at pH 6.7. The pH values in this study ranged from 6.63 to 6.82. However, the difference was not statistically significant. This study is in line with Okoruwa and Aidelomon [25], who found that the supplementation of sheep and goat diets with ginger powder had no significant effect on the pH value of the rumen with the supplementation of ginger powder in sheep and goat diets. The insignificant differences in pH values in the present study show that the experimental diets could provide a pH balance in the rumen fluid to support the microbial ecosystem. In addition, the production of VFA and NH3 affects the pH of rumen fluid. An increase in VFA will cause a decrease in rumen fluid pH, whereas an increase in NH_3_ will cause an increase in rumen fluid pH. The pH range between 6.63 and 6.82, obtained in this study, is still within the normal rumen pH range of 5.5–7.0 [42]. In this study, addition of herbal had no effect on rumen fluid pH; therefore, the fermentative activity of rumen microorganisms was not disturbed.

### Partial VFA and total VFA concentration

The VFA concentrations, including those of acetate, propionate, and butyrate, were examined in this study. VFAs are end products of carbohydrate and protein fermentation. Volatile fatty acids are a source of energy for ruminants. The concentrations of acetate, propionate, butyrate, and total VFAs in this study are listed in Table-5. The results of the analysis showed that the total VFA, propionate, and butyrate acid concentrations in the present study were not significantly different from the control (p > 0.05). In contrast, acetate acid concentration was significantly different (p < 0.05). The mean total VFA concentration in this study ranged from 34.99 mM to 44.71 mM. A previous study by McDonald *et al*. [36] stated that the optimum total VFA concentration to support the activity of microbial rumen is 70 Mm–150 Mm. The previous study reported by Ramandhani *et al*. [44] that adding papaya and *C. longa* Linn. Leaf extracts containing saponins to dairy cow rations increased the total VFA from 162.5 to 445 mMol. Cherdthong *et al*. [45] found that total VFA was not altered by supplementing *Piper sarmentosum*, a traditional medicine and food flavoring agent widely abundant in Thailand, in cattle diet. The high concentration of total VFA illustrates the high amount of organic matter in the diet that is easily degraded by rumen microbes. A high VFA concentration is associated with balanced rumen pH, which allows rumen microbes to function properly.

The average total VFA concentration in this study was still below the normal values. This was presumably due to the long incubation time, which exceeded the optimum time for increasing VFA. The length of the incubation time results in a reduced availability of feed ingredients to be fermented by microbes, resulting in a decreased rate of VFA production as an indication of reduced energy availability for ruminants. We assumed that low VFA concentration in this study was due to the secondary metabolites from herbal plants. Similarly, a previous study by Jayanegara *et al*. [46] stated that there is an inverse correlation between tannin and total VFA and that tannin can reduce rumen fermentation. Several factors affect VFA concentration, including the physical form of the feed, the type of dissolved carbohydrates, rumen pH, digestibility of feed ingredients, feed basalt, and the addition of secondary chemical metabolites in the diet.

The partial VFA concentration is influenced by the feed composition. The production of acetate, propionate, and butyrate acids depends on carbohydrate fermentation, particularly non-fiber carbohydrates (NFC) [47]. The process of forming acetate and butyrate acids produces H_2_ and CO_2_ which are used by methanogen bacteria for the formation of CH_4_. The higher the acetate and butyrate acids, the higher was the CH_4_ production. Saponins found in herbal plants can increase the number of rumen bacteria, total VFA, and the levels of acetic acid, propionate acid, and NH_3_.

### Average gas production and gas production rate per hour

The results showed that there was a significant effect (p < 0.05) of herbal plants on the average gas production (Table-6), while there was an insignificant effect on the gas production rate per hour (p > 0.05). The addition of herbal plants, namely, *M. oleifera*, *C. longa* Linn., and *P. timoriana*, or their combination increased the total gas production compared to the control treatment (p < 0.05). This shows that supplementation with herbal plants can increase microbial activity in the rumen. The increased microbial activity in the rumen enhances the fermentation of feed nutrients. Fermentation of NFCs results in high gas production due to increased fermentation process [48]. This is consistent with a previous study by Faniyi *et al*. [11] that found a significant difference in gas production when the diet was supplemented with herbal plants.

The highest average gas production was observed in the R7 treatment, namely, the supplementation with a combination of *C. longa* Linn. and *P. timoriana*. The lowest total gas production was observed in R2 treatment, namely, supplementation with *M. oleifera*. Total gas production was lower in the R8 treatment (combination of *M. oleifera*, *C. longa* Linn., and *P. timoriana*) than in the R7 treatment but higher than the other treatments (p < 0.05). This indicates an increase in gas production with the supplementation of a combination of herbal plants. Increased gas production is due to the presence of saponin-active compounds in herbal plants; therefore, rumen bacteria work well in degrading the rumen. In this study, the gas production rate per hour was not significantly different between the experimental treatment (p > 0.05). This indicates that the *in vitro* gas production rate decreased with increasing incubation time due to the fermentable substrate [49].

### Methane gas emissions and protozoa populations

Methane is produced during anaerobic fermentation of feed ingredients in the rumen by methanogenic bacteria and reflects the loss of feed energy. As shown in Table-7, the supplementation of herbal plants reduced CH_4_ gas (p < 0.05). This is because propionate acid production utilizes more H_2_, whereas acetate and butyrate acid production produce H_2_. Thus, the increase in propionate acid decreased CH_4_ production. CH_4_ gas concentrations were not significantly different between the experimental treatments including either one or a combination of herbal plants. This was presumably due to the fixed concentration of herbal plants (30%). Thus, the levels of active substances contained after the addition of one or a combination of herbal plants were comparable.

The higher the amount of CH_4_ gas produced, the more inefficient the feed. Herbal plants contain saponins, flavonoids, tannins, alkaloids, and phenolic compounds [31]. The saponin and tannin contents of herbal plants can affect rumen characteristics by modulating rumen fermentation. These secondary metabolites reduce methanogen populations by reducing protozoa associated with methanogens or directly inhibiting methanogens [50]. The decrease in CH_4_ gas production is directly proportional to the decrease in the protozoan population because the availability of H_2_ for methanogens decreases, and a decrease in the protozoa population in the rumen results in a higher population of bacteria [49]. In addition, protein turnover in the rumen is lower and the amount of microbial protein that enters the duodenum increases [50, 51].

The results showed that the control treatment (R1) with the addition of one of the herbal plants or a combination (R2, R3, R4, R6, R7, and R8) did not significantly reduce protozoan population. In contrast, Cherdthong *et al*. [45] found that the supplementation of *P. sarmentosum* in Thai native beef cattle diet significantly reduced the protozoan population, which altered the reduction of CH_4_ production. They stated that the flavonoid compounds present in *P. sarmentosum* suppressed methanogenesis by reducing the protozoan population, thus reducing methanogenic bacteria that are symbiotic with protozoa. The present study found no significant effects on the protozoan population. Based on CH_4_ production and protozoa populations, it can be concluded that protozoa populations in the rumen are not the dominant factor in the process of methanogenesis. In support, it has been shown that a small proportion of methanogens attaches to the surface of protozoan cells; therefore, the role of protozoa in methanogenesis is not dominant [50, 52].

## Conclusion

Supplementation with a combination of *M. oleifera* and *C. longa* Linn. effectively increased the percentage of IVDMD and IVOMD, which were more easily degraded, increased the total gas production rate, and reduced CH4 gas emissions and protozoan populations during rumen fermentation compared with the control treatment. This study investigated the effects of herbal plants as feed supplements for ruminants on nutrient digestibility and rumen fermentation products using *in vitro* method. Follow-up research using *in vivo* method is required to evaluate the effectiveness of herbal plants as feed supplements to increase ruminant productivity and reduce CH_4_ gas production.

## Authors’ Contributions

AA and RP: Designed the study and reviewed the manuscript. AA, NL, MR, SS, and AJ: Conducted field and laboratory work and data tabulation. YM, SA, LM, EMP, and WN: Conducted the data analysis and drafted and revised the manuscript. All authors have read, reviewed, and approved the final manuscript.
